# Examining population structure of a bertha armyworm, *Mamestra configurata* (Lepidoptera: Noctuidae), outbreak in western North America: Implications for gene flow and dispersal

**DOI:** 10.1371/journal.pone.0218993

**Published:** 2019-06-27

**Authors:** Martin A. Erlandson, Boyd A. Mori, Cathy Coutu, Jennifer Holowachuk, Owen O. Olfert, Tara D. Gariepy, Dwayne D. Hegedus

**Affiliations:** 1 Saskatoon Research and Development Centre, Agriculture and Agri-Food Canada, Saskatoon, SK CANADA; 2 London Research and Development Centre, Agriculture and Agri-Food Canada, London, ON CANADA; National Cheng Kung University, TAIWAN

## Abstract

The bertha armyworm (BAW), *Mamestra configurata*, is a significant pest of canola (*Brassica napus* L. and *B*. *rapa* L.) in western North America that undergoes cyclical outbreaks every 6–8 years. During peak outbreaks millions of dollars are spent on insecticidal control and, even with control efforts, subsequent damage can result in losses worth millions of dollars. Despite the importance of this pest insect, information is lacking on the dispersal ability of BAW and the genetic variation of populations from across its geographic range which may underlie potential differences in their susceptibility to insecticides or pathogens. Here, we examined the genetic diversity of BAW populations during an outbreak across its geographic range in western North America. First, mitochondrial cytochrome oxidase 1 (*CO1*) barcode sequences were used to confirm species identification of insects captured in a network of pheromone traps across the range, followed by haplotype analyses. We then sequenced the BAW genome and used double-digest restriction site associated DNA sequencing, mapped to the genome, to identify 1000s of single nucleotide polymorphisms (SNP) markers. *CO1* haplotype analysis identified 9 haplotypes distributed across 28 sample locations and three laboratory-reared colonies. Analysis of genotypic data from both the *CO1* and SNP markers revealed little population structure across BAW’s vast range. The *CO1* haplotype pattern showed a star-like phylogeny which is often associated with species whose population abundance and range has recently expanded and combined with pheromone trap data, indicates the outbreak may have originated from a single focal point in central Saskatchewan. The relatively recent introduction of canola and rapid expansion of the canola growing region across western North America, combined with the cyclical outbreaks of BAW caused by precipitous population crashes, has likely selected for a genetically homogenous BAW population adapted to this crop.

## Introduction

Dispersal is an essential life history trait as it affects survival, reproduction, colonization of new environments and, ultimately, gene flow within and between populations [1]. Depending on the connectivity of populations, and hence the ability of organisms to move between them, gene flow may be inhibited leading to localized adaptations or uninhibited which would increase gene flow and dilute local adaptions [2–4]. This is of particular concern in agricultural environments where pest species are often under strong selective pressure due to crop rotations, changing crop varieties, and the application of control products (e.g. herbicides, insecticides, fungicides) which may result in selection of detrimental traits (e.g. insecticide resistance) [5, 6] that could lead to increased crop losses. In order to understand population connectivity, the dispersal ability of organisms and gene flow among individuals, direct approaches including mark-release-recapture, electronic tags and radar-tracking, and indirect approaches which include examination of range expansion records and laboratory flight behaviour experiments have been undertaken [1]. However, monitoring movement and dispersal of organisms in the field is often difficult and impractical to observe [1, 7]. Population genetics provides a platform to infer the movement and gene flow of individuals among different sampled localities through the use of genetic markers (e.g. mitochondrial haplotypes, microsatellites, single nucleotide polymorphism (SNPs) [8, 9] and references therein) and has been widely used to track invasions of non-native species and infer dispersal patterns in a wide range of insect pests [3, 10–12].

**Fig 1:**
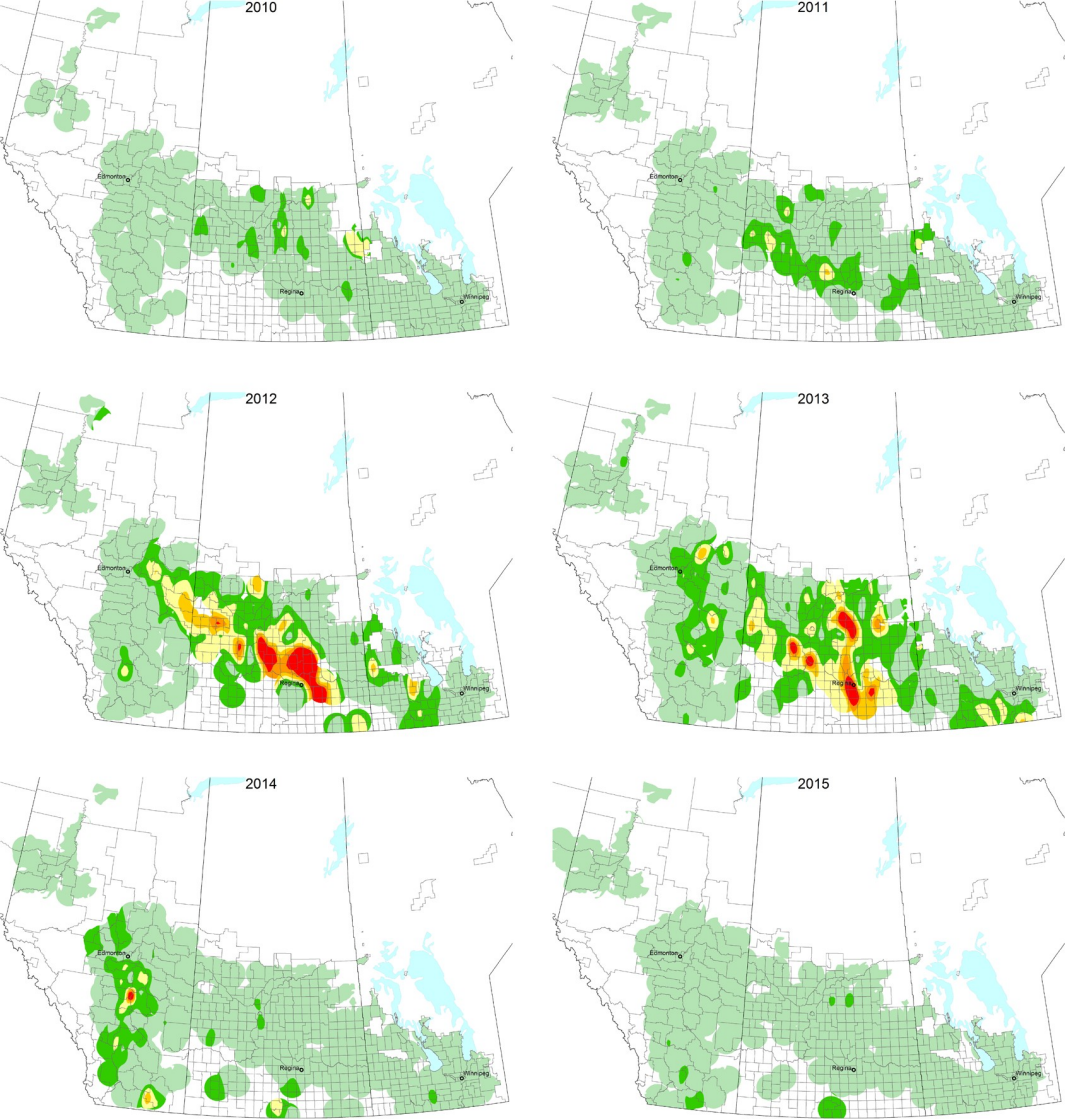
Season long pheromone trap collection data for *Mamestra configurata* during the 2010–2015. The colour of localities indicate cumulative moth counts; 0–300 (pale green), 300–600 (dark green), 600–900 (yellow), 900–1200 (pale orange), 1200–1500 (dark orange) and 1500+ (red).

In species that undergo cyclical outbreaks, dispersal plays an important role in synchronizing population dynamics and genetic similarity [13, 14]. The bertha armyworm (BAW), *Mamestra configurata* Walker (Lepidoptera: Noctuidae), is native to western Canada and feeds on a wide range of plant species [15, 16]; however, it also undergoes cyclical outbreaks on canola. Canola, *Brassica napus* L. and *B*. *rapa* L. (*Brassicaceae*), is a domesticated oilseed-crop of Mediterranean origin [17] and is grown on over 9 million ha, contributing more than $26.7 billion (CAD) to the Canadian economy [18, 19]. Larvae feed on foliage and developing seedpods, negatively affecting seed quality and causing substantial seed yield losses [16]. Across western Canada, major regional outbreaks of BAW occur every 6–8 years and last up to 4 years [16]. The scale of insecticide spray application can be significant when BAW outbreaks occur. In two recent outbreaks (1994–1996 and 2005–2007), between 600,000 and 800,000 ha of canola were sprayed annually at cost of approximately $16.5 million CAD [16, 20]. Estimated yield losses ranged from $10 to 40 million CAD annually despite these control efforts. The latest BAW outbreak across the Canadian prairie provinces started in 2011 and persisted until 2014 (Fig 1).

The factors that drive the outbreak cycles of BAW populations are not completely understood. This is particularly the case with respect to the conditions required for the increase in populations at the beginning of an outbreak cycle. As resident BAW populations can be found across the Prairies in non-outbreak years, it is hypothesized that outbreaks are driven by favorable environmental conditions that lead to build up of local populations from one year to the next. From a population genetics perspective, if outbreaks are driven by local populations, we expect there would be evidence of population structure across the range of BAW. Alternatively, expanding populations in consecutive years of the outbreak cycle may result from the migration of founding moth populations from epicenters of resurgence. Little is known about the migratory/dispersal ability of BAW, but circumstantial evidence based on experiments to test pheromone and light trapping efficiency concluded that moths dispersed beyond the outer ring of traps placed up to 200 m from the release point [21]. In addition, Swailes et al. [22] found traps baited with virgin females captured males as far as 80 km from previously known infestations, suggesting moths can disperse up to 80 km and possibly more. If outbreaks are driven by the dispersal of migrants from a single focal point, then we would expect little population structure as migrants would homogenize genetic diversity across the range. While it is not known whether outbreaks begin via a buildup of local populations or spread from an epicenter, a few factors have been implicated in the seemingly precipitous crash of outbreak populations. Epizootics of baculoviruses (Mamestra configurata nucleopolyhedrovirus-A and -B) and entomopathogenic fungi have been associated with massive collapses of late instar BAW larval populations in the field and it is thought that these diseases are major mortality factors that eventually curtail BAW outbreaks [16, 23–25]. In addition, native parasitoids appear to contribute to the regulation of BAW populations [26].

Despite the importance of this pest insect, little is known about the dispersal ability of BAW or the genetic variation of populations from across its geographic range which may underlie potential differences in their susceptibility or response to chemical insecticides or pathogens. Prior to the start of the current study, only seven BAW cytochrome oxidase 1 (*CO1*) barcode sequences were deposited in the Barcode of Life Data System (BOLD) (http://www.barcodinglife.org/) from which two potential haplotypes could be identified. Thus, we undertook an extensive sampling strategy to collect male BAW moths from across its geographic range during the latest outbreak in collaboration with the “Prairie Insect Pest Monitoring Network” (PIPMN) annual pheromone trap monitoring program which is used to determine BAW population abundance (http://www.westernforum.org/IPMNMain.html). These male moths were used to undertake *CO1* haplotype analysis and to develop restriction site associated DNA sequencing (RAD-seq) libraries for high throughput sequencing and SNP marker identification. The aims of the research described herein were to; i) develop genomics resources for BAW including a whole genome assembly, ii) to generate a panel of SNP markers to investigate BAW genetic diversity and population structure across its geographic range in western North America during an outbreak, and thus infer dispersal ability, and iii) to determine whether long term BAW laboratory colonies reflect the genetic content of wild BAW populations.

## Results

### Genome assembly and annotation

A draft genome for BAW was assembled to use as a reference for the ddRAD-seq analysis and SNP identification. The assembled genome was 571.3 Mb, and comprised 86,779 scaffolds with an N50 of 207.7 kb. Fully, 98.6% of the DNA reads mapped onto the genome. BUSCO analysis using the Endopterygota_odb9 gene set indicated that 2,012 (82.4%) of the core genes from Endopterygota were present and full length, a further 198 (8.1%) were present and partial, and 232 (9.5%) were missing. Only 24 (1.1%) of the full length genes were duplicated. The 571.3 Mb assembled genome size compared favorably with the size estimated using flow cytometry which ranged between 590.9 ±10.6 Mb in males (N = 5) to 607.4 ± 2.9 Mb in females (N = 5). The genome has been deposited in the NCBI (accession # NDFZ00000000) and I5K (http://i5k.github.io/genomes) databases. The assembly statistics for the current version of the BAW genome compares favourably with that of other Noctuidae genome sequences (S1 Table).

### Mitochondrial CO1 analyses

During the 2012 through 2014 growing seasons, ~5,100 male BAW moths were collected from pheromone-baited traps placed in canola fields across the western Canadian provinces of Manitoba, Saskatchewan, Alberta and British Columbia under the auspices of the PIPMN. These years captured the end of the most recent BAW outbreak cycle which began in 2011 (Fig 1). A random sample of ~ 450 individuals, representing various geographic collections sites from across the three provinces were selected for *CO1* barcode sequencing. The *CO1* barcode sequence analysis showed that at least 10 other Lepidoptera: Noctuidae taxa were collected in small numbers in BAW pheromone traps including: 26 *Apamea cogitata* Smith, 1 *Apamea commoda* Walker, 5 *Apamea devastator* (Brace), 1 *Chersotis juncta* (Grote), 37 *Enargia spp*. Hübner, 7 *Euxoa spp*. Hübner, 3 *Feltia jaculifera* (Guenée), 3 *Leucania anteroclara* Smith, 10 *Mniotype spp*. Franclemont, 1 *Peridroma saucia* (Hübner) and 2 *Xestia smithii* (Snellen).

**Fig 2:**
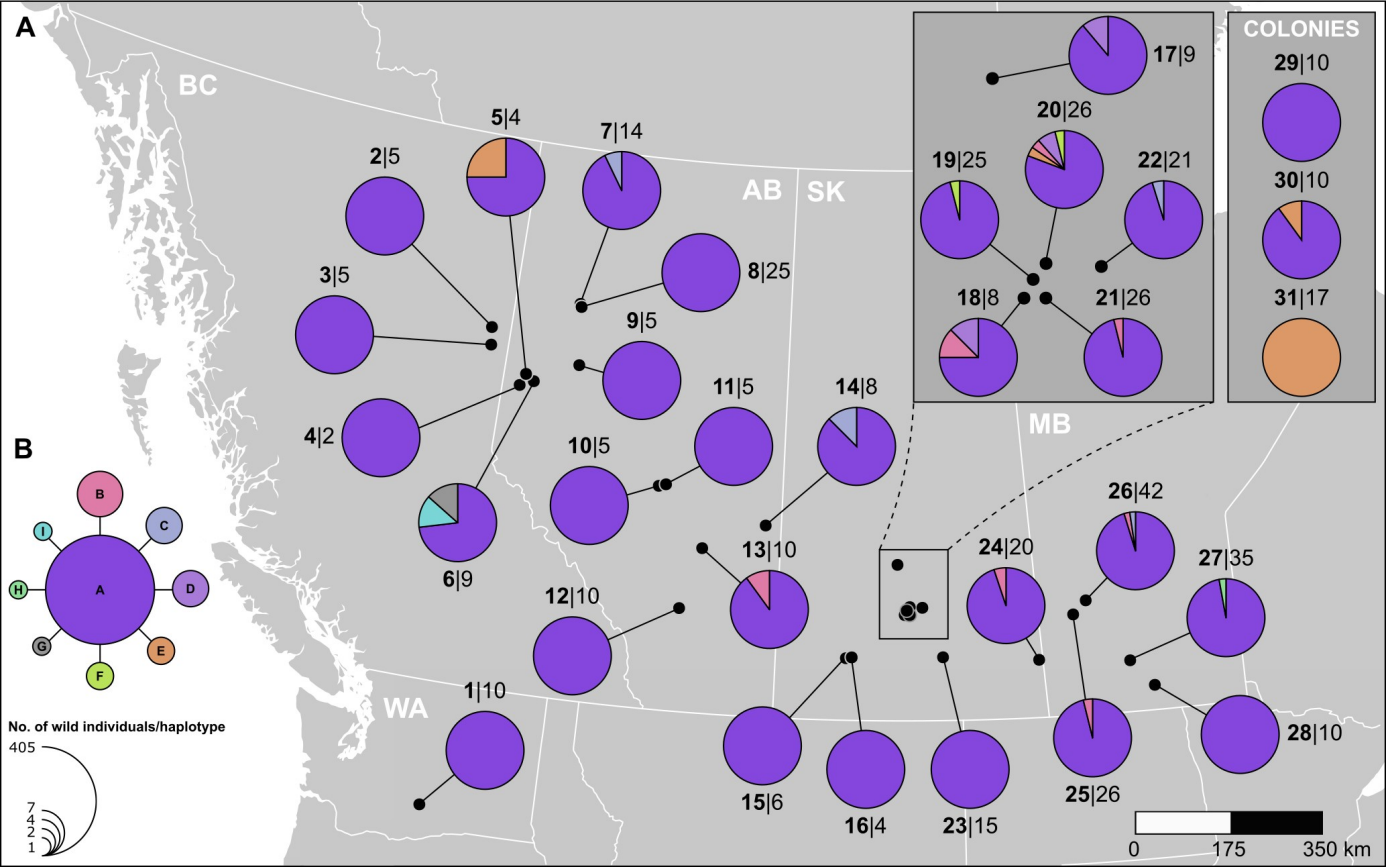
Haplotype analysis of mitochondrial *CO1* sequences from *Mamestra configurata* male from wild and laboratory cultures. (A) Geographic distribution and haplotype prevalence for *M*. *configurata* individuals from 28 collection sites across western North America and 3 laboratory colonies. Bold *numbers* prior to | indicate sample locality (corresponds to Table 1) and the *numbers* following | indicates the sample size. Each color in the pie charts corresponds to a different haplotype and corresponds to haplotype *letters* in panel B. (B) Haplotype network for the 9 identified haplotypes in the wild BAW populations with corresponding *letters*. Size of the nodes is proportional to the number of individuals with each haplotype. Each connection denotes a single mutational step.

From among the specimens confirmed to be BAW by *CO1* barcode sequencing (GenBank Accession #s MH880344—MH880788), a total of 9 haplotypes were observed throughout 28 sampling localities in Washington State, western Canada, and 3 AAFC laboratory colonies (Table 1, Fig 2A). The *CO1* haplotype analysis revealed a star-like phylogeny with a single dominant haplotype from which a small number of minor haplotypes radiate (Fig 2B). The single dominant haplotype (A) was found in all sampling localities except the AAFC Saskatoon colony. The long-term AAFC colony was fixed for haplotype E, whereas the AAFC Davidson colony (13 generations in colony) had individuals of both haplotype A and E, and the short-term AAFC Lethbridge colony (2 generations in colony) was fixed for haplotype A. Three haplotypes were represented by only a single individual (G, H, I) and these were collected near the periphery of the sampled range. The highest haplotype and nucleotide diversity occurred in the central Saskatchewan localities of Girvin and Imperial, while those locations at the western, northern, and eastern most periphery of the range sampled (Wapato, WA, Manning, AB, Carman, MB, respectively) had the lowest diversity (Table 1, Fig 2A). Tajima’s *D* was negative (*D* = –1.85) and significantly different from 0 (*p* < 0.001) when all wild samples were combined, which signifies an excess of low frequency polymorphisms across the sampling localities and suggests a recent, rapid population expansion [27]. AMOVA detected significant population structure with groups (colony and wild) accounting for most of the variation (60.9%) (Table 2) which was most likely driven by the large number of individuals fixed for haplotype E in the AAFC Saskatoon colony. Populations within the groups accounted for a small percentage (11.1%) of the genetic variation, while a larger percentage (28%) occurred within populations (Table 2). When wild populations, grouped by region, were subjected to AMOVA no source of variation was statistically significant (Table 2). Pair-wise *F*_*ST*_ comparisons were generally low and non-significant across all sampling localities indicating little population differentiation and no population structure, the exception being the long-term AAFC Saskatoon colony which was significantly different from all wild and other colony populations (Table 3). There was no evidence of isolation-by-distance (IBD) in wild populations (*y* = 9*e*^−6^*x* + 0.0228, r^2^ = 0.002, *p* > 0.05).

**Table 1:** Locality, geographic and haplotype information for samples used in *CO1* haplotype analysis including haplotype distribution and diversity statistics.

Locality	State/Province	Regionǂ	Site Name	Latitude	Longitude	Haplotypes (*n*)	Haplotype Diversity (Mean ± SD)	Nucleotide Diversity (Mean ± SD)
1*	Washington	Washington	Wapato	46.471	-120.381	A (10)	0.0000 ± 0.0000	0.0000 ± 0.0000
2*	British Columbia	Peace Region	Baldonnel	56.245	-120.689	A (5)	0.0000 ± 0.0000	0.0000 ± 0.0000
3*	British Columbia	Peace Region	Farmington	55.898	-120.610	A (5)	0.0000 ± 0.0000	0.0000 ± 0.0000
4*	Alberta	Peace Region	Beaverlodge	55.195	-119.393	A (20)	0.0000 ± 0.0000	0.0000 ± 0.0000
5	Alberta	Peace Region	La Glace	55.433	-119.231	A (3), E (1)	0.5000 ± 0.2652	0.0012 ± 0.0013
6	Alberta	Peace Region	Clairmont	55.312	-118.923	A (7), I (1), G (1)	0.4167 ± 0.1907	0.0009 ± 0.0010
7	Alberta	Peace Region	Notikewin	56.967	-117.664	A (13), C (1)	0.1429 ± 0.1188	0.0003 ± 0.0005
8*	Alberta	Peace Region	Manning	56.908	-117.610	A (25)	0.0000 ± 0.0000	0.0000 ± 0.0000
9*	Alberta	Peace Region	Girouxville	55.756	-117.417	A (5)	0.0000 ± 0.0000	0.0000 ± 0.0000
10*	Alberta	Central Alberta	Carvel	53.578	-113.971	A (5)	0.0000 ± 0.0000	0.0000 ± 0.0000
11*	Alberta	Central Alberta	Stony Plain	53.534	-114.210	A (5)	0.0000 ± 0.0000	0.0000 ± 0.0000
12*	Alberta	Central Alberta	Hilton	51.104	-113.180	A (10)	0.0000 ± 0.0000	0.0000 ± 0.0000
13*	Alberta	Central Alberta	Viking	52.354	-112.584	A (9), B (1)	0.2000 ± 0.1541	0.0004 ± 0.0006
14*	Alberta	Central Alberta	Wainwright	52.895	-110.524	A (7), C (1)	0.2500 ± 0.1802	0.0005 ± 0.0007
15*	Saskatchewan	Saskatchewan	Swift Current	50.262	-107.749	A (6)	0.0000 ± 0.0000	0.0000 ± 0.0000
16*	Saskatchewan	Saskatchewan	Rosenhof	50.281	-107.563	A (4)	0.0000 ± 0.0000	0.0000 ± 0.0000
17*	Saskatchewan	Saskatchewan	St. Denis	52.173	-106.111	A (8), D (1)	0.2222 ± 0.1662	0.0005 ± 0.0007
18	Saskatchewan	Saskatchewan	Girvin	51.154	-105.874	A (6), B (1), D (1)	0.4643 ± 0.2000	0.0011 ± 0.0011
19	Saskatchewan	Saskatchewan	Davidson	51.241	-105.806	A (24), F (1)	0.0800 ± 0.0722	0.0002 ± 0.0004
20	Saskatchewan	Saskatchewan	Imperial	51.315	-105.710	A (21), B (1), D (2), E (1), F (1)	0.3508 ± 0.1172	0.0008 ± 0.0009
21*	Saskatchewan	Saskatchewan	Liberty	51.154	-105.712	A (25), B (1)	0.0769 ± 0.0697	0.0002 ± 0.0004
22	Saskatchewan	Saskatchewan	Eller's Beach	51.300	-105.300	A (20),C (1)	0.0952 ± 0.0843	0.0002 ± 0.0004
23*	Saskatchewan	Saskatchewan	Bratt's Lake	50.288	-104.652	A (15)	0.0000 ± 0.0000	0.0000 ± 0.0000
24*	Saskatchewan	Saskatchewan	Moosomin	50.174	-101.596	A (19), B (1)	0.1000 ± 0.0880	0.0002 ± 0.0004
25	Manitoba	Manitoba	Gilbert Plains	51.059	-100.426	A (25), B (1)	0.0769 ± 0.0697	0.0002 ± 0.0004
26*	Manitoba	Manitoba	Sifton	51.328	-99.990	A (40), B (1), C (1)	0.0941 ± 0.0611	0.0002 ± 0.0004
27*	Manitoba	Manitoba	MacGregor	50.042	-98.710	A (34), H (1)	0.0571 ± 0.0532	0.0001 ± 0.0003
28*	Manitoba	Manitoba	Carman	49.499	-98.001	A (10)	0.0000 ± 0.0000	0.0000 ± 0.0000
29*	Colony	NA	AAFC Lethbridge	49.682	-112.780	A (10)	0.0000 ± 0.0000	0.0000 ± 0.0000
30*	Colony	NA	AAFC Davidson	51.300	-105.713	A (9), E (1)	0.2000 ± 0.1541	0.0004 ± 0.0006
31*	Colony	NA	AAFC Saskatoon	52.134	-106.635	E (17)	0.0000 ± 0.0000	0.0000 ± 0.0000

ǂ Geographic region used to form groups for AMOVA analyses. Groups formed based on geographic isolation and geopolitical boundaries.

*Denotes localities from which samples for ddRAD-seq analysis were selected

NA = not applicable

**Table 2:** Analysis of molecular variance (AMOVA) results on mitochondrial *CO1* and SNP data.

		*CO1*			SNPs		
Group	Source of variation	*d*.*f*.	Variation (%)	*p* value	*d*.*f*.	Variation (%)	*p* value
Colony vs. wild	Among groups	1	60.88	< 0.0001	1	4.57	< 0.0001
	Among populations within groups	29	11.1	< 0.0001	21	3.47	< 0.0001
	Within populations	414	28.02	0.023	397	91.95	0.009
Region*	Among groups	4	-0.42	*ns*	4	0.01	*ns*
	Among populations within groups	23	0.42	*ns*	15	-0.12	*ns*
	Within populations	380	100	*ns*	330	100.1	*ns*

*Region corresponds to region in Table 1.

*ns* = not significant

**Table 3:** Pair-wise *F*_*ST*_ estimates for *CO1* sequences (below diagonal) and SNP analysis (above the diagonal).

Site Name	Locality	1	2	3	4	5	6	7	8	9	10	11	12	13	14	15	16	17	18	19	20	21	22	23	24	25	26	27	28	29	30	31
Wapato	1		0.008	0.007	—	—	**0.008**	**—**	**0.008**	0.009	0.005	0.003	**0.008**	0.004	0.003	**0.009**	0.006	**0.007**	**—**	**—**	**—**	0.003	**—**	**0.007**	**0.006**	**—**	**0.009**	**0.014**	0.005	**0.138**	**0.216**	**0.244**
Baldonnel	2	0.000		0.008	—	—	0.008	**—**	0.001	0.000	0.000	0.000	0.000	0.000	0.003	0.000	0.000	0.007	**—**	**—**	**—**	0.001	**—**	0.000	0.000	**—**	0.000	0.009	0.000	**0.155**	**0.279**	**0.320**
Farmington	3	0.000	0.000		—	—	0.005	**—**	0.000	0.000	0.000	0.000	0.000	0.000	0.001	0.000	0.007	0.007	**—**	**—**	**—**	0.000	**—**	0.003	0.001	**—**	0.001	0.011	0.000	**0.162**	**0.292**	**0.359**
Beaverlodge	4	0.000	0.000	0.000		—	—	**—**	**—**	**—**	**—**	**—**	**—**	**—**	**—**	**—**	**—**	**—**	**—**	**—**	**—**	**—**	**—**	**—**	**—**	**—**	**—**	**—**	**—**	**—**	**—**	**—**
La Glace	5	0.245	0.063	0.063	0.434		—	**—**	**—**	**—**	**—**	**—**	**—**	**—**	**—**	**—**	**—**	**—**	**—**	**—**	**—**	**—**	**—**	**—**	**—**	**—**	**—**	**—**	**—**	**—**	**—**	**—**
Clairmont	6	0.012	0.000	0.000	0.097	0.009		**—**	0.002	0.002	0.006	0.005	0.005	0.002	0.000	0.005	0.005	0.005	**—**	**—**	**—**	0.002	**—**	0.002	**0.004**	**—**	0.005	0.010	**0.006**	**0.128**	**0.200**	**0.226**
Notikewin	7	0.000	0.000	0.000	0.026	0.139	0.024		**—**	**—**	**—**	**—**	**—**	**—**	**—**	**—**	**—**	**—**	**—**	**—**	**—**	**—**	**—**	**—**	**—**	**—**	**—**	**—**	**—**	**—**	**—**	**—**
Manning	8	0.000	0.000	0.000	0.000	0.495	0.128	0.044		0.004	0.003	0.002	0.002	0.004	0.000	0.000	0.007	0.002	**—**	**—**	**—**	0.001	**—**	0.003	0.001	**—**	0.003	0.008	0.000	**0.124**	**0.192**	**0.215**
Girouxville	9	0.000	0.000	0.000	0.000	0.063	0.000	0.000	0.000		0.000	0.001	0.003	0.002	0.000	0.000	0.006	0.000	**—**	**—**	**—**	0.000	**—**	0.000	0.000	**—**	0.000	0.004	0.000	**0.142**	**0.235**	**0.273**
Carvel	10	0.000	0.000	0.000	0.000	0.063	0.000	0.000	0.000	0.000		0.000	0.003	0.001	0.000	0.005	0.004	0.005	**—**	**—**	**—**	0.000	**—**	0.004	0.000	**—**	0.003	0.009	0.006	**0.141**	**0.239**	**0.278**
Stony Plain	11	0.000	0.000	0.000	0.000	0.063	0.000	0.000	0.000	0.000	0.000		0.000	0.000	0.004	0.001	0.002	0.000	**—**	**—**	**—**	0.000	**—**	0.000	0.000	**—**	0.000	0.002	0.001	**0.136**	**0.233**	**0.274**
Hilton	12	0.000	0.000	0.000	0.000	0.245	0.012	0.000	0.000	0.000	0.000	0.000		0.002	0.002	0.002	0.002	0.001	**—**	**—**	**—**	0.001	**—**	0.000	0.003	**—**	0.002	0.010	0.000	**0.128**	**0.208**	**0.228**
Viking	13	0.000	0.000	0.000	0.075	0.081	0.005	0.005	0.102	0.000	0.000	0.000	0.000		0.000	0.000	0.006	0.001	**—**	**—**	**—**	0.000	**—**	0.001	0.000	**—**	0.001	0.005	0.000	**0.122**	**0.199**	**0.229**
Wainwright	14	0.029	0.000	0.000	0.126	0.050	0.000	0.000	0.163	0.000	0.000	0.000	0.029	0.003		0.000	0.001	0.000	**—**	**—**	**—**	0.000	**—**	0.000	0.000	**—**	0.000	0.006	0.000	**0.140**	**0.240**	**0.279**
Swift Current	15	0.000	0.000	0.000	0.000	0.111	0.000	0.000	0.000	0.000	0.000	0.000	0.000	0.000	0.000		0.007	0.000	**—**	**—**	**—**	0.000	**—**	0.002	0.001	**—**	0.001	0.007	0.000	**0.128**	**0.218**	**0.250**
Rosenhof	16	0.000	0.000	0.000	0.000	0.000	0.000	0.000	0.000	0.000	0.000	0.000	0.000	0.000	0.000	0.000		0.002	**—**	**—**	**—**	0.001	**—**	0.000	0.000	**—**	0.000	0.008	0.004	**0.147**	**0.261**	**0.304**
St. Denis	17	0.012	0.000	0.000	0.097	0.066	0.000	0.009	0.128	0.000	0.000	0.000	0.012	0.001	0.001	0.000	0.000		**—**	**—**	**—**	0.000	**—**	0.001	0.000	**—**	0.001	0.007	0.001	**0.128**	**0.198**	**0.222**
Girvin	18	0.029	0.000	0.000	0.126	0.000	0.001	0.036	0.163	0.000	0.000	0.000	0.029	0.000	0.000	0.000	0.000	0.000		**—**	**—**	**—**	**—**	**—**	**—**	**—**	**—**	**—**	**—**	**—**	**—**	**—**
Davidson	19	0.000	0.000	0.000	0.000	0.265	0.072	0.010	0.000	0.000	0.000	0.000	0.000	0.032	0.059	0.000	0.000	0.043	0.095		**—**	**—**	**—**	**—**	**—**	**—**	**—**	**—**	**—**	**—**	**—**	**—**
Imperial	20	0.000	0.000	0.000	0.004	0.000	0.014	0.000	0.014	0.000	0.000	0.000	0.000	0.000	0.000	0.000	0.000	0.000	0.000	0.000		**—**	**—**	**—**	**—**	**—**	**—**	**—**	**—**	**—**	**—**	**—**
Liberty	21	0.000	0.000	0.000	0.000	0.275	0.077	0.011	0.000	0.000	0.000	0.000	0.000	0.000	0.063	0.000	0.000	0.046	0.052	0.000	0.001		**—**	0.002	0.000	**—**	0.002	0.006	0.000	**0.126**	**0.200**	**0.228**
Eller's Beach	22	0.000	0.000	0.000	0.000	0.224	0.056	0.000	0.009	0.000	0.000	0.000	0.000	0.022	0.000	0.000	0.000	0.031	0.075	0.001	0.007	0.001		**—**	**—**	**—**	**—**	**—**	**—**	**—**	**—**	**—**
Bratt's Lake	23	0.000	0.000	0.000	0.000	0.355	0.060	0.005	0.000	0.000	0.000	0.000	0.000	0.043	0.084	0.000	0.000	0.060	0.084	0.000	0.000	0.000	0.000		0.001	**—**	0.004	0.007	0.003	**0.111**	**0.169**	**0.171**
Moosomin	24	0.000	0.000	0.000	0.000	0.213	0.051	0.004	0.011	0.000	0.000	0.000	0.000	0.000	0.040	0.000	0.000	0.028	0.015	0.001	0.000	0.000	0.000	0.000		**—**	0.000	0.006	0.000	**0.111**	**0.153**	**0.163**
Gilbert Plains	25	0.000	0.000	0.000	0.000	0.275	0.077	0.011	0.000	0.000	0.000	0.000	0.000	0.000	0.063	0.000	0.000	0.046	0.052	0.000	0.001	0.000	0.001	0.000	0.000		**—**	**—**	**—**	**—**	**—**	**—**
Sifton	26	0.000	0.000	0.000	0.000	0.276	0.093	0.000	0.000	0.000	0.000	0.000	0.000	0.000	0.017	0.000	0.000	0.047	0.088	0.000	0.016	0.000	0.000	0.000	0.000	0.000		0.006	0.001	**0.110**	**0.161**	**0.173**
MacGregor	27	0.000	0.000	0.000	0.000	0.351	0.111	0.023	0.000	0.000	0.000	0.000	0.000	0.057	0.096	0.000	0.000	0.073	0.141	0.002	0.024	0.000	0.005	0.000	0.006	0.002	0.000		0.008	**0.121**	**0.193**	**0.230**
Carman	28	0.000	0.000	0.000	0.000	0.245	0.012	0.000	0.000	0.000	0.000	0.000	0.000	0.000	0.029	0.000	0.000	0.012	0.029	0.000	0.000	0.000	0.000	0.000	0.000	0.000	0.000	0.000		**0.122**	**0.188**	**0.209**
AAFC Lethbridge	29	0.000	0.000	0.000	0.000	0.245	0.012	0.000	0.000	0.000	0.000	0.000	0.000	0.000	0.029	0.000	0.000	0.012	0.029	0.000	0.000	0.000	0.000	0.000	0.000	0.000	0.002	0.000	0.000		**0.346**	**0.394**
AAFC Davidson	30	0.000	0.000	0.000	0.075	0.000	0.005	0.005	0.102	0.000	0.000	0.000	0.000	0.000	0.003	0.000	0.000	0.001	0.012	0.032	0.000	0.034	0.022	0.043	0.019	0.034	0.034	0.057	0.000	0.000		**0.468**
AAFC Saskatoon	31	**1.000**	**1.000**	**1.000**	**1.000**	**0.875**	**0.872**	**0.940**	**1.000**	**1.000**	**1.000**	**1.000**	**1.000**	**0.933**	**0.930**	**1.000**	**1.000**	**0.931**	**0.870**	**0.954**	**0.800**	**0.955**	**0.950**	**1.000**	**0.948**	**0.955**	**0.936**	**0.963**	**1.000**	**1.000**	**0.918**	

**Bold text** indicates significant differences (FDR corrected *p* < 0.05)

—indicates samples not included in ddRAD-seq dataset

### SNP analyses

Multiplexed libraries from individual moths were sequenced across 5 lanes on the Illumina HiSeq 2500 platform resulting in over 1.4 billion reads, of which 322 million remained after filtering (average number of reads = 1.35 million/individual). Paired-end reads (average 1.2 million/individual) were aligned to the BAW reference genome, which led to 127,233 loci being called from the *populations* pipeline in Stacks. De-multiplexed and filtered reads were deposited in NCBI Sequence Read Archive (SRP158535).

SNP loci were genotyped in all individuals with a high degree of success across the populations (mean 3074 SNPs/individual). Allelic richness was relatively low in wild populations (1.199–1.209) and even lower in colony populations (1.037–1.126). Observed heterozygosity varied little across populations from wild localities (0.095–0.102); however, it was lower in most colony populations (0.034–0.087) (Table 4). In particular, the AAFC Davidson colony (13 generations in culture) and the AAFC Saskatoon colony (>100 generations) had the lowest level of genetic diversity, likely related to longer durations of “inbreeding” in these closed colonies, but with random mating (Table 4). *F*_*IS*_ values were negative for all localities indicating that individuals were less related than would be expected under random mating; however, none were considered significantly different from 0 based on permutation tests. Private alleles were only found in the three AAFC colony populations and each colony contained between 5 to 13 private alleles (S2 Table), combined with low heterozygosity (Table 4) this lead to high measures of pairwise differentiation between the colonies and the wild populations (Table 3). Within the wild populations, population differentiation was low and generally non-significant indicating little genetic population structure (Table 3). Only 3 loci, all from the AAFC Saskatoon colony population, significantly deviated from Hardy-Weinberg equilibrium (HWE)(after FDR correction); however, due to the extremely low number of loci they were not removed from the analyses (S3 Table). In contrast to the *CO1* analysis, AMOVA indicated that a small percentage of variation was accounted for among groups (4.6%) when wild and colony populations were compared, and the majority of the variation occurs within populations (91.3%) (Table 2). When wild populations were compared by region, no source of variation was significant (Table 2). As with the *CO1* data, there was no IBD in wild populations when the SNP dataset was assessed (*y* = 0.0013*x* − 0.0009, r^2^ = 0.02, *p* > 0.05).

**Table 4:** Allelic richness (*Ar*), Heterozygosity (*Ho*, *He*) and *F*_*IS*_ inbreeding coefficient estimates of *Mamestra configurata* populations subjected to ddRAD-seq analysis.

Locality	Site Name	*n*	*Ar*	*Ho*	*He*	*F*_IS_*
1	Wapato	10	1.208	0.100	0.094	-0.037
2	Baldonnel	5	1.205	0.100	0.085	-0.152
3	Farmington	3	1.209	0.100	0.082	-0.008
6	Clairmont	9	1.204	0.100	0.093	-0.070
8	Manning	10	1.208	0.098	0.094	0.006
9	Girouxville	5	1.205	0.096	0.089	0.021
10	Carvel	5	1.214	0.102	0.092	-0.001
11	Stony Plain	5	1.199	0.095	0.086	0.017
12	Hilton	8	1.206	0.099	0.093	-0.008
13	Viking	10	1.208	0.100	0.093	-0.166
14	Wainwright	7	1.211	0.102	0.091	-0.137
15	Swift Current	6	1.206	0.097	0.091	0.015
16	Rosenhof	4	1.206	0.100	0.087	-0.001
17	St. Denis	9	1.200	0.097	0.091	-0.008
21	Liberty	8	1.209	0.099	0.094	-0.027
23	Bratt's Lake	15	1.212	0.101	0.098	-0.005
24	Moosomin	19	1.209	0.099	0.098	-0.001
26	Sifton	17	1.206	0.099	0.097	-0.004
27	MacGregor	10	1.201	0.098	0.093	-0.057
28	Carman	10	1.212	0.100	0.096	-0.009
29	AAFC Lethbridge	8	1.126	0.087	0.067	-0.311
30	AAFC Davidson	10	1.055	0.047	0.040	-0.275
31	AAFC Saskatoon	17	1.037	0.034	0.032	-0.238

*No *F*_*IS*_ values are significantly different from 0 according to bootstrapped 95% C.I.

**Fig 3:**
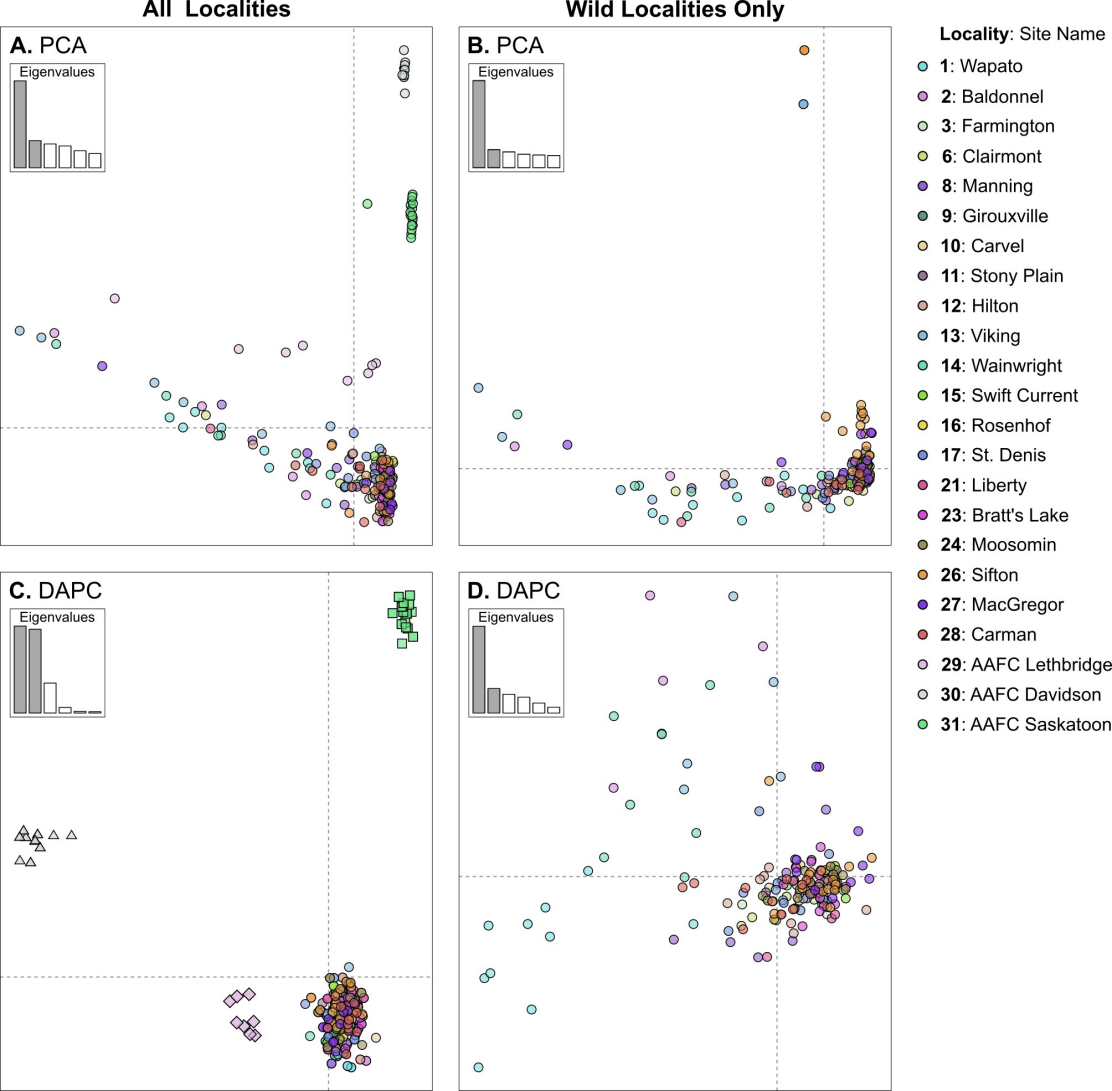
Results of the ordination based analyses conducted on SNP BAW dataset. (A) Principles component analysis (PCA) conducted on all localities (wild and colonies). (B) PCA conducted on wild localities only. (C) Discriminate analysis of principle components (DAPC) conducted on all localities (wild and colonies). Point shape corresponds to genetic cluster. (D) DAPC conducted on wild localities only. Color of points in all panels corresponds to collection locality. Inset plots within panels indicates relative contributions of individual eigenvalues of the top six principle components, A and B, or discriminate functions, C and D.

When colony and wild individuals were included in the PCA analysis only the three colony populations (localities 29–31) were differentiated from the others (Fig 3A). No differentiation was observed when only wild individuals were examined (Fig 3B). In addition, when DAPC was conducted on both wild and colony individuals the *find*.*cluster* function predicted a value of *K* = 4, which corresponded to a separate genetic cluster for each colony and a single genetic cluster composed of all the wild individuals (Fig 3C). There was limited population differentiation when wild individuals were examined separately (Fig 3D). Although, the *find*.*cluster* function indicated *K* = 2 the vast majority of the variance is separated on the first discriminate axis and additional clusters were not clearly differentiated (Fig 3D).

**Fig 4:**
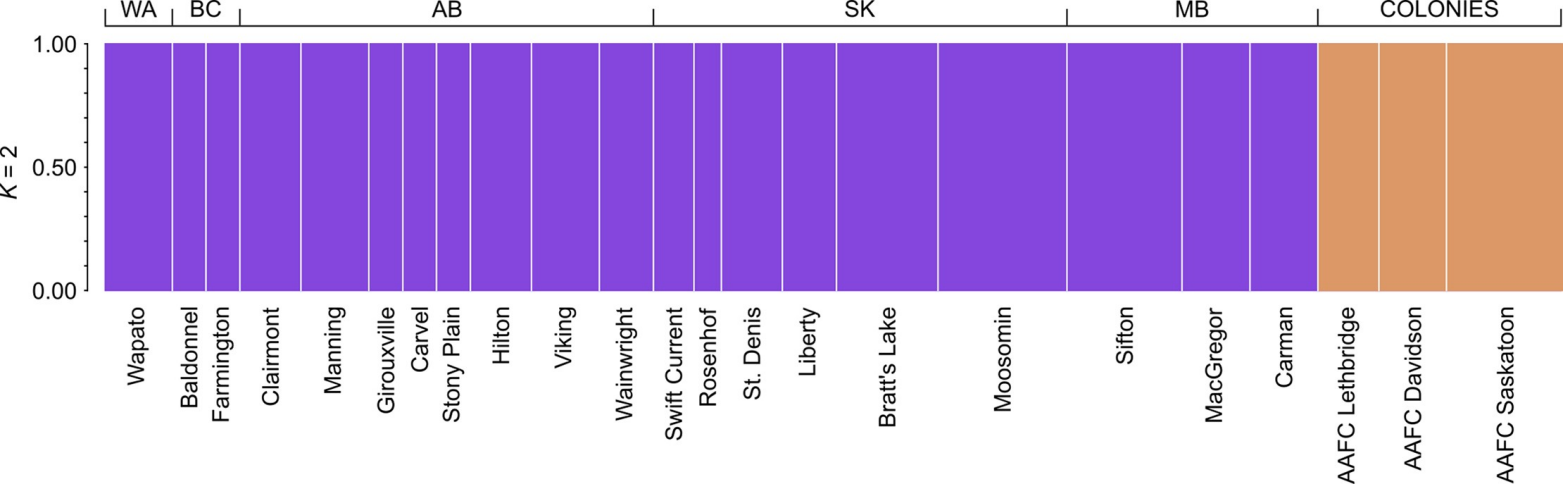
Bayesian assignment probabilities based on BAW SNP data as inferred by STRUCTURE. Each individual is represented by a single column divided into *K* genetic clusters. Assignment of all individual samples (n = 210) from the whole data (wild and colony) set to *K* = 2 genetic clusters. Province/State of collection localities are given for each individual above the plot, and the locality name below the plot. Localities are separated by vertical white lines.

When STRUCTURE was run on all localities including the wild and colonies the most likely number of genetic clusters was *K* = 2 (Fig 4). Ln Pr(*X*|*K*), delta *K*, and the median and medians and means all supported *K* = 2 whereas the maximum of medians and means supported *K* = 4 (S1 Fig). However, when the STRUCTURE results were examined at *K* = 4 no additional structure was observed (S2 Fig). As with the PCA and DAPC, no structure was observed when STRUCTURE was run on the wild individuals only assuming admixture, correlated allele frequencies and with sampling location specified (locprior model) [28]. Across the sampled localities the most likely number of genetic clusters was *K* = 1 (S3 and S4 Figs).

## Discussion

Bertha armyworm has been a major insect pest in western Canada dating back to the early 20^th^ century and was first noted in flax in the 1920s [15, 16]. However, massive and widespread cyclic outbreaks have occurred from the 1950s onwards concomitant with the introduction of *B*. *rapa* and *B*. *napus* varieties of oilseed rape and later canola. There is limited information on the pre-agriculture distribution and host plant range of BAW; however, based on its polyphagous nature, having been recorded from at least 40 host plant species, its major native host plant range is thought to have been various species within the Chenopodiaceae and the Brassicaceae [16]. In recent years (1995 onwards), data has been collected on its distribution and abundance via an extensive pheromone trap system across western Canada established and managed under the auspices of the PIPMN. However, almost nothing is known about its genetic diversity across it geographic range or its dispersal ability. The current study is the first to address genetic diversity of BAW populations and provides a snapshot of the genetic makeup of populations at the peak of an outbreak.

### Genome assembly

To assist with the population genetics study, a BAW genome assembly was generated based on three sibling BAW males from a long-term (ca. 35 years) laboratory colony (AAFC Saskatoon). The 571.3 Mb genome assembly represents the largest noctuid genome assembled to date and the assembly statistics (eg. scaffold N_50_ = 2.1 x 10^5^ bp) were in line with other noctuid species that have been sequenced recently, including *Spodoptera frugiperda* [29], *Spodoptera litura* [30], *Helicoverpa zea* and *Heliothis virescens* [31] that range in size from 341 to 438 Mb and with scaffold N_50_s from 1.0 x 10^5^ to 1.0 x 10^6^ bp. Recently, high quality genome assemblies have been generated for *Helicoverpa armigera* [31] and *Trichoplusia ni* [32], the assembly of the later is notable for its virtually chromosome-length scaffold assemblies. The current BAW genome assembly proved useful for mapping informative SNPs for the population genetics analysis and is currently being used extensively for transcriptomics analysis of various BAW tissues and developmental stages.

### Population structure

Analyses using both mitochondrial *CO1* and SNP genotyping revealed little population differentiation across the range of BAW in western North America. A low number of *CO1* haplotypes were observed and comprised a haplotype network with a classic “star phylogeny” with a dominant (91% of individuals) haplotype at the core and a small number of minor haplotypes representing single nucleotide changes. This haplotype pattern is often associated with species whose population abundance and range has recently, in evolutionary time scale, undergone an expansion [33]. This is supported by the negative Tajima’s *D* statistic (*D* = –1.85) which indicates the presence of low frequency polymorphisms among individuals from the various sampled localities [27]. Pair-wise *F*_*ST*_ based on *CO1* sequence and SNP comparisons were generally low and non-significant across all wild sampling localities indicating no population structure. In addition, the SNP-based AMOVA determined that the majority of genetic variation is attributed to the individual level in wild populations. All these results, along with the lack of IBD and weak to no population differentiation suggests that BAW populations have undergone a recent expansion and has moved widely across its range.

In addition, the results of the PCA, DAPC and STRUCTURE analyses suggest that the wild BAW populations had as much variation within local populations as across the entire BAW population. These types of observations often occur with wild populations that are not limited by geographic barriers, as is the case for a large area of BAW geographic distribution, resulting in a continuous population arising from extensive genetic transfer between adjacent/sympatric populations. STRUCTURE often predicts the occurrence of a single population when sampling is uniform across landscapes and individuals are examined *en masse* [34]. This pattern would seem to fit with the recent and relatively sudden availability of highly suitable host plants with the introduction of *B*. *rapa* and *B*. *napus* varieties of oilseed rape and then canola within the agricultural context of western Canada and the ensuing observations of cyclic outbreaks of BAW.

### Dispersal ability and outbreaks

Population genetics provides a platform to infer gene flow and dispersal ability of insects which is often difficult to obtain directly in the field [1, 7]. BAW is no exception, and little information is available on its dispersal ability. Circumstantial evidence suggests BAW can disperse beyond 200 m and as far as 80 km [21, 22]. These distances are not inconceivable, given that several noctuid moths including *Spodoptera litura* (Fabricius), *S*. *exempta* (Walker), *S*. *frugiperda* (J. E. Smith) and *Helicoverpa zea* (Boddie) are estimated to disperse over 10–20 km/day [35, 36] and several hundred kilometers over their life [37, 38]. Surprisingly, no BAW population structure was observed across wild populations over an area of ca. 1 million km^2^ including a somewhat isolated population west of the Rocky Mountains in Wapato, Washington. The lack of population structure indicates that the distribution of BAW at the time of this study arose from the same panmictic population. Lack of population structure has been noted in several outbreaking species, including on continental scales [10, 39] and even across island populations [13]. Studies on outbreak and cyclic populations of small mammals [40] (and references therein) and insects [13] have generally found that the movement of individuals across the landscape is sufficient to homogenize local populations [41]. The ability of BAW to disperse over a wide geographic range may have implications if a strong selection event resulted in a dominant trait that is detrimental to crop production (e.g. insecticide or pathogen resistance) as it would spread quickly across the range. However, the reverse is also true as immigration of BAW into the local population would mask and dilute the trait if a recessive detrimental trait arose in an isolated population. Examining the spread of the last BAW outbreak (Fig 1) and the distribution of *CO1* haplotypes, it appears that the epicentre of the outbreak originated in east central Saskatchewan (Girvin and Imperial) which was the region of highest BAW density and greatest *CO1* haplotype diversity (Fig 2; Table 1). Movement of moths containing haplotype A and E to the west, combined with moths from haplotype B and C moving both east and west could result in the distribution observed. The singleton haplotypes observed at the periphery of the distribution in Clairmont, Alberta (haplotype G, I) and in MacGregor, Manitoba (haplotype H) may have occurred *de novo* at these sites, or they could be the remnants of the historical haplotypes that were overwhelmed by the current outbreak.

James *et al*. [14] demonstrated that the time of sample collection during an outbreak will have direct consequences on the subsequent population structure, with samples taken at the peak of outbreaks having lower *F*_*ST*_ and, hence, higher homogeneity than those taken at the rise or decline of the outbreak (troughs). They further suggest that during peak outbreaks over several thousands of kilometers several scenarios may be occurring in which some populations at peak outbreak have low genetic structure, while others are at intermediate or low population levels have intermediate or high degrees of spatial genetic structure [14]. BAW doesn’t appear to support this later scenario as no spatial genetic structure was observed across its range even in areas outside of the peak outbreak regions. Further studies are necessary to determine the spatial genetic structure at different times of the BAW outbreak cycle (e.g. in the rise or decline phase).

Several insect herbivores have been noted to maintain diversity on ancestral host species, but genetic diversity was reduced when range expansions occurred following introduction of a new domesticated crop [42–44]. Therefore, it should be noted that isolated BAW populations may still exist that are not associated with canola and are, therefore, not adapted to this host plant. These may be genetically distinct from the dominant outbreak population present in the more extensive canola growing areas of the Canadian prairies and should be more apparent in non-outbreak years, though very few moths are collected at these times.

### Laboratory colony diversity compared to wild populations

Laboratory colonies are ideal resources to conduct behavioural and physiological studies on crop pests. The colonies can be maintained and individuals produced in regular intervals, preventing the need to wait for the appropriate seasons in order to collect insects from the field. However, the selection of individuals for colonies may lead to adaptations that are not present in the wild and thus results with laboratory colonies may not directly transfer to wild populations. For this reason, we compared the genetic diversity of AAFC-maintained laboratory colonies of BAW with those captured in the wild. The short-term (Lethbridge and Davidson) and long-term (Saskatoon) laboratory colonies were moderately differentiated from wild populations based on pairwise *F*_*ST*_ comparisons (0.110–0.359). This conclusion was also borne out by the PCA and DAPC analyses which showed that the Davidson AAFC colony and the long-term Saskatoon AAFC colony populations, 13 and >100 generations in culture respectively, clearly separated from the wild populations (Fig 3). The individuals from the Lethbridge AAFC colony, 1^st^ generation in culture, were also somewhat differentiated from the bulk of the wild individuals. STRUCTURE also supported a separate cluster for the colonies compared to the wild populations (Fig 4). Significantly, the laboratory colonies were the only populations that contained private alleles and the observed heterozygosity values, *Ho*, were substantially lower than that in the wild populations. These results are similar to those observed in a genetic comparison of field populations, long-term colonies, selected breeding colonies and colonies derived from 10 generations of sib-mating in *H*. *virescens* [45]. Although the method of SNP selection and genetic diversity estimations were somewhat different in Fritz et al. [45] compared to the current study, both studies showed that most of the genetic diversity of field populations was present within populations as opposed to between populations. An earlier study of North American *H*. *virescens* genetic diversity based on AFLP markers also showed that 98% of total genetic diversity occurred within populations and the geographically-distributed populations were quite homogenous [46]. The other conclusion in common between the current study and that of Fritz et al. [45] was the substantially lower observed heterogeneity (*Ho* values) in established laboratory colonies and higher pairwise *F*_*ST*_ estimates for laboratory colony vs wild populations than among wild populations, again suggesting reduced genetic diversity in the colony populations and genetic divergence from the wild populations. These results have implications if studies are conducted on colony individuals only, as adaptations to the laboratory may lead to results not indicative of wild populations.

## Conclusions

The relatively recent introduction of canola and rapid expansion of the canola growing region across the prairies, combined with the cyclical outbreaks of BAW caused by precipitous population crashes, has likely selected for a genetically homogenous BAW population adapted to this crop. The cause of the increase in populations during an outbreak is difficult to ascertain, but it is most likely caused by a reduction in natural enemies (e.g. predators, parasites, and pathogens) after BAW populations crash, combined with favourable environmental parameters, which allow the pest populations to rebuild. Regardless of what controls BAW population cycles, data from BAW sampling acquired by the PIPMN through several outbreaks, along with the results presented here, appears to show that outbreaks originate from one or a few focal points, expand outward and then collapse accordingly.

## Material and methods

### Haploid genome size estimations by propidium iodide nuclear staining

The BAW genome size was estimated using flow cytometry [47]. Briefly, individual heads of a single male or female adult BAW were placed in 1 ml of Galbraith buffer along with the head of a female *Drosophila virilis* as an internal standard (1C = 328 Mb genome). The two heads were co-ground with a pestle in a 1 ml Dounce grinder, filtered through a 20 micron nylon mesh and stained with 0.25 mg of propidium iodide (PI). The mean PI fluorescence of the stained co-prepared nuclei from the sample and standard were scored using a CyFlow (Partec USA) flow cytometer. To ensure that only nuclei, free of cellular tags, were used in the assay a gate was set on side scatter and only nuclei with uniformly low side scatter (small uniform size) were scored for fluorescence. A minimum of 1000 gated diploid nuclei were scored for both the standard and the sample. The total DNA amount was determined as the ratio: (average channel number of the sample 2N/ average channel number of the standard 2N) multiplied by the 1C amount of DNA in the standard.

### Genome sequencing and assembly

An initial genome assembly for BAW was conducted for use as a reference genome to which RAD-seq reads could be aligned for SNP identification. Briefly, DNA extracted from 3 male siblings from a long-established (> 35 years in culture), highly inbred laboratory colony (AAFC Saskatoon) was used to generate 6 Illumina paired-end genomic DNA libraries (insert sizes ranging from 300 bp to 10 kb) and 4 Roche 454 paired-end genomic DNA libraries (insert sizes ranging from 15–40 kb). These libraries were sequenced at the National Research Council, Plant Biotechnology Institute (Saskatoon, Saskatchewan, Canada) by Illumina HiSeq or Roche 454 FLX-titanium pyrosequencing, respectively. All reads were trimmed for adapters and quality, and duplicate and orphaned reads were removed using Trimmomatic v.0.3.0 [48]. Mitochondrial DNA reads and PhiX reads were identified and removed using CLC Genomics Workbench v.8.0.3 **(**https://www.qiagenbioinformatics.com/), and error correction using SOAPec v.2.01 [49] was applied to all remaining reads. The genome was assembled using SOAPdenovo2 v.2.04-r240 [49], with a kmer of 63 for contig assembly and 47 for scaffold assembly. Gaps between contigs were filled or partially filled by running two iterations of GapCloser v.1.12 [49] on the assembled scaffolds. Seventy draft genome assemblies were generated, and the best was chosen by balancing the scaffold N50, contig N50, total size and average gap length. SOAPdenovo settings that were tested included Kmer size (39–85), order of library incorporation, coverage required to join contigs and gap length estimation. Read pre-treatments that were tried included: error correction (SOAPec v2.02), removal of PhiX (which included mapping reads to PhiX genome using CLC genomics workbench and then keeping reads which did not map), removal of mitochondrial reads and removal of potential contaminants (DeconSeq v.0.4.3) [50]. Small scaffolds that were >95% identical to portions of larger scaffolds were removed using CLC Genomics Workbench and scaffolds less than 500 bp long were also removed.

Additionally, the quality of the genome assembly was assessed using BUSCO v.3.0.2 against the endopterygota_odb9 dataset (Creation date: 2016-02-13, number of species: 35, number of BUSCOs: 2,442) [51, 52].

### Insects

Male BAW moths were collected from pheromone traps from across western Canada as part of the PIPMN annual monitoring program for BAW population abundance (http://www.westernforum.org/IPMNMain.html). The moths were captured during the most recent BAW outbreak from 2011–2014 (Fig 1). Male moths were also collected by individual collaborators using light traps or other baited trap systems. Moths were stored frozen at -80°C until processing for DNA extraction.

### DNA extraction protocol for *CO1* PCR

For mitochondrial *CO1* gene haplotype investigations only, DNA was extracted from the two hind legs using a DNeasy Blood and Tissue Kit (Qiagen, Toronto, Ontario, Canada) following the manufacturer’s protocol for insect tissue. The DNA was eluted in a final volume of 50 μL (elution buffer AE) and concentrations were estimated using a NanoDrop 2000 spectrophotometer (Thermo Fisher Scientific, Burlington, Ontario, Canada).

### *CO1* PCR Amplicon Sequencing

BAW mitochondrial *CO1* gene sequences were amplified using the barcoding primers LCO1490-F (GGTCAACAAATCATAAAGATATTGG) and HCO2198-R (TAAACTTCAGGGTGACCAAAAAATCA) [53] with the following thermal cycling conditions: an initial denaturation step at 94°C for 1 min followed by 5 cycles at 94°C for 40 s, 45°C for 40 s, and 72°C for 1 min; 35 cycles at 94°C for 40 s, 51°C for 40 s, 72°C for 1 min; and 72°C for 5 min. Resulting *CO1* amplicons were separated by electrophoresis on agarose gels and purified using a QIAquick Gel Extraction Kit (Qiagen) and sequenced at the National Research Council, Plant Biotechnology Institute (Saskatoon, Saskatchewan, Canada). The resulting mitochondrial *CO1* sequences were assembled, aligned and trimmed using SeqMan Pro v. 13 (DNASTAR, Madison, Wisconsin, USA). The final *CO1* dataset included sequences from 445 individuals, 227 individuals for which only *CO1* sequence was obtained and 218 individuals processed for *CO1* sequence and ddRAD-seq SNP data (S4 Table).

### Whole insect DNA extraction and ddRAD-seq library construction

Male moths (n = 218) from 23 localities from across western NA were selected for ddRAD-seq analysis (Table 1; S4 Table) and genomic DNA extracted using the following procedure. Heads and wings were removed from individual moths, and the remaining tissues were homogenized in 1.5 mL microcentrifuge tubes in 700 μL of Lifton buffer (200 mM sucrose, 50 mM Na-EDTA, 100 mM Tris-HCl, 0.5% SDS) using a nylon pestle. After homogenization, an additional 300 μL of Lifton buffer and 25 μL of proteinase K (5 mg/mL) were added and the samples incubated at 65°C for 120 min. The samples were then centrifuged at 14,000 *g* for 10 min, after which the supernatant was removed to a new 1.5 mL microcentrifuge tube. Twenty-five μL of RNAse A (10 mg/mL) was added to the samples and they were incubated at RT for 5 min. Finally, 100 μL of 8 M potassium acetate was added, the suspension was mixed using a vortex, incubated on ice for 30 min and then centrifuged at 14,000 *g* for 15 min. The supernatant (~800 μL) was removed to a new 1.5 mL microcentrifuge tube and the DNA extracted with the addition of 400 μL of phenol:chloroform:isoamyl alcohol (25:24:1). The aqueous phase was clarified by centrifugation at 14,000 *g* for 8 min and then transferred to a new tube. Chloroform was used to extract the aqueous phase, and then the sample was clarified by centrifugation at 14,000 *g* for 8 min, after which the aqueous phase was transferred to a new tube. The DNA was precipitated by the addition of 500 μL of isopropanol and was pelleted by centrifugation at 14,000 *g* for 20 min. The isopropanol was removed and the DNA pellet washed with 300 μL of 70% ethanol. The DNA pellet was air-dried for 10 min at RT after which it was resuspended in 100 μL of sterile ddH_2_O. Genomic DNA concentrations and purity were initially assessed using a NanoDrop 2000 spectrophotometer and finally by fluorescence using PicoGreen (Invitrogen Quant-iT dsDNA assay kit) and a Perkin-Elmer VICTOR X2 fluorometer.

The ddRAD-seq approach was adapted from Poland *et al*. [54] using the DNA restriction enzymes *Pst*I and *Msp*I. All reactions were carried out in 96-well plates in groups of 48 individual DNA samples. DNA samples were diluted to 20 ng/μL and 10 μL added to each well along with 2 μL of 10 X NEB Buffer #4, 0.4 μL *Pst*I-HF (8 units), 0.4 μL *Msp*I (8 units) and 7.2 μL ddH_2_O. The plates were incubated at 37°C for 2 h for DNA digestion, 65°C for 20 min for restriction enzyme inactivation and then held at 8°C. The same plate was then processed for a ligation step by the addition of 5 μL of an adapter mix (0.02 μM of the unique barcode adapter, Adapter 1, and 3 μM the of the common Y-adapter, Adapter 2), 2 μL of 10 X NEB Buffer #4, 4 μL ATP (1 mM), 0.5 μL T4 DNA ligase (200 U) and 8.5 μL ddH_2_O. The plate was incubated at 22°C for 2 h and then at 65°C for 20 min for ligation and ligase enzyme inactivation and then held at 8°C. A series of 8 PCR reactions were performed using 10 μL from each of 48 sample ligations pooled into a single 1.5 mL tube and purified using a QIAquick PCR Purification Kit (Qiagen). Multiple PCR reactions were run for each library to minimize the impact of any random amplification bias that may occur in a single PCR reaction. Each PCR reaction included 10 μL of DNA (ligation pool), 5 μL 5 X NEB Master Mix, 2 μL Illumina PE forward and reverse primers (10 uM) and 8 μL ddH_2_O. The PCR thermocycler conditions were as follows: 95°C for 30 sec, 16 cycles of 95°C for 30 sec, 62°C for 20 sec, 68°C for 30 sec, a final extension at 72°C for 5 min, and then held at 4°C. The short extension time for the PCR reaction cycles was designed to enrich for fragments of 150–300 bp. A QIAquick PCR Column Purification Kit was used to purify 200 μL of the pooled ligation PCR mixture by adding 1.0 mL of PB buffer and adding sequential 600 μL volumes to the column with subsequent centrifugation steps. The ligation pool PCR reactions for 48 individual pools were then resuspended in 30 μL of elution buffer, evaluated for fragment size distribution using an Agilent Bioanalyzer 2100 (Agilent Technologies, Mississauga, Ontario, Canada). The ddRAD-seq libraries for 218 BAW individuals were partitioned over and sequenced on 5 Illumina HiSeq2500 lanes.

### SNP marker identification

Stacks v.2.0b [55, 56] was used to filter reads, process loci and genotype individuals. First, *process_radtags* was used to demultiplex raw paired-end FASTQ files according to unique barcode adapters, remove reads with Illumina adapter contamination, uncalled bases, and low quality scores, and correct barcode and restriction enzyme cut sites (default parameters). The cleaned reads were then aligned to the BAW genome (NCBI accession # NDFZ00000000) using default settings with the Burrow-Wheelers aligner v.0.7.17 [57] and the MEM algorithm [58]. Next, the Stacks script *ref_map*.*pl* was used to execute the Stacks components *gstacks* and *populations*. *Gstacks* assembles loci, creates a catalog and calls SNPs for each individual based on a maximum-likelihood method (low sequencing coverage parameter–model Marukilow) [59]. Finally, *populations* was used to group all individuals as a single “population” with loci needing to be present in 1% (parameter–r 0.01) of individuals to be included in the final VCF formatted output file.

The *populations* VCF output file was further filtered with VCFTools v.0.1.15 [60]. First, a minimum read depth of 5 was specified in order to process a locus for an individual. Next, missing data per individual was calculated after first removing loci missing >20% genotype data in order to remove individuals contributing to high missing data at particular loci. Eight individuals were removed from the *populations* VCF formatted output file based on the above criteria. Next, the remaining individuals (n = 210) were re-filtered to remove loci with >20% missing genotype data, with a minor allele frequency of < 1%, and that were not in Hardy-Weinberg equilibrium (HWE). To avoid using SNPs that maybe linked and possibly in a state of linkage disequilibrium, loci were thinned so that SNPs were at least 50 kbp apart. All the filtering resulted in a final dataset containing 3,353 SNPs in 210 individuals (7–109 x coverage) (S5 and S6 Tables).

### Assessment of genetic diversity

#### i) Mitochondrial DNA—*CO1*

In order to assess genetic diversity among individuals, haplotype and nucleotide diversity [61] were calculated in Arlequin v.3.5.2.2 [62] and the relationships among haplotypes were examined by constructing a maximum parsimony haplotype network with a 95% connection limit with TCS v. 1.21 [63, 64]. To test for changes in demographic history across BAW populations, a test of neutrality, Tajima’s D [27], was calculated in Arlequin. Tajima’s D can be used as an indication of a recent population expansion (e.g. following a bottleneck) when the null hypothesis of neutrality is rejected due to significant negative values, whereas significant positive values would indicate a population contraction [27].

#### ii) SNPs

To explore genetic diversity in the SNP dataset, allelic richness (*A*_*R*_), observed (*H*_*O*_) and expected heterozygosity (*H*_*E*_), were calculated with the ‘diveRsity’ package [65] in R v.3.5.0 [66] and inbreeding coefficients (*F*_*IS*_) (with 1023 bootstrap replicates) were calculated in Arlequin. In addition, locus-specific deviations from Hardy-Weinberg equilibrium (HWE) were tested in GenoDive v.2.ob27 (least-squares AMOVA method, 10,000 permutations) [67] and the number of private alleles across loci were calculated using the ‘poppr’ package v.2.8.0 [68, 69] in R.

### Assessment of population structure

To investigate population differentiation, pairwise-*F*_*ST*_ values were calculated between individual sampling localities (populations) based on *CO1* haplotypes (pairwise difference based method with 10,000 permutations) in Arlequin and SNPs (using the AMOVA-based method with 10,000 permutations) in GenoDive. A false discovery rate (FDR) correction procedure was applied to all tests (*CO1* and SNP) with multiple comparisons [70]. In addition, population structure was examined with hierarchical analysis of molecular variance (AMOVA) [71]. In order to determine if significant genetic differentiation occurs between laboratory colonies and wild populations, the effect of source of individuals (laboratory colony vs. wild) on genetic differentiation was examined. Next, to determine if collection region led to significant genetic differentiation among wild populations, the effect of geographic region (Washington State, Peace Region of British Columbia and Alberta, Central Alberta, Saskatchewan and Manitoba) was examined. Geographic regions were selected based on geographic isolation and geopolitical boundaries due to clustering of sample localities within these regions. AMOVA was conducted in Arlequin (10,100 permutations) using both the *CO1* and SNP dataset.

As distance alone can sometimes influence genetic differentiation between populations, isolation-by-distance (IBD) [72] was assessed between all wild populations through the regression of matrices of standardized genetic distance (*F*_*ST*_/1-*F*_*ST*_) [73] and geographical distance (log_10_ transformed) [74]. The significance of correlation between the standardized genetic and geographic distances was evaluated with a Mantel test [75] in GenAlx v. 6.5 [76]. Geographic distance between sampling localities was calculated with the Geographic Distance Matrix Generator v. 1.2.3 [77].

Finally, in order to infer the number of genetic clusters and assess population structure two different approaches were taken: i) model-free ordination techniques including a multivariate principal components analysis (PCA) and a discriminate analysis of principle components (DAPC) [78–80], and; ii) a model-based, Bayesian algorithm, clustering approach in STRUCTURE v.2.3.4 [81]. The data input into the PCA was based on the individuals and their SNP allele frequencies, and summarizes the overall genetic variability among individuals across all SNP loci, but does not assess groups/clusters. PCA was first performed on all populations to determine if colonies were differentiated from wild populations. A second PCA was used to identify possible population structure with the wild populations only. DAPC differs from PCA in that it aims to maximize between group variation and minimize within group variation, in doing so DAPC identifies the optimal number of genetic clusters represented in the data. For the DAPC, the function *find*.*clusters* was used in order to estimate *K*, the most probable number of distinct genetic clusters, while retaining all principle components (PCs). In order to optimize the number of PCs to retain in the final DAPC analysis, a cross-validation procedure (*xvalDapc* function) was used, with 50 replicates and 200 PCs maximum. PCA and DAPC were conducted with the ‘adegenet’ package v.2.0 [78–80] in R. As with the PCA, DAPC was first conducted on all populations, and subsequently the wild populations alone.

In addition to the ordination-based approaches, STRUCTURE, which uses a Bayesian approach that minimizes HWE and linkage disequilibrium within clusters, was used to infer the most probable number of distinct genetic clusters (*K*) across the sampled localities [81]. Initially, all populations were compared using the no admixture model, and independent allele frequencies with twenty iterations of K = 1–15. No admixture and independent allele frequencies were used in the analysis as there was no possibility of admixture between laboratory colony and wild populations. A second analysis was conducted on wild populations only, and consisted of twenty iterations of K = 1–15 with an admixture model, correlated allele frequencies [82], and with sample localities as priors [28]. Runs consisted of a burn-in period of 50,000 replicates, followed by 150,000 Monte Carlo Markov Chain replicates. CLUMPAK [83] was used to average results across iterations and STRUCTUREselector [84] which implements Ln P(*K*) [81], delta *K* [85] and median of means, maximum of means, median of medians and maximum of medians [86] was used to select the most likely value of *K*.

## Supporting information

S1 Fig Results of best K selection from STRUCTURE for all individuals (wild and colony individuals) combined. A) Ln Pr(X|*K*). B) Delta *K*. C) Median of medians and means. D) Maximum of medians and means.
(TIFF)

S2 Fig CLUMPAK results of STRUCTURE run on all individuals (wild and colony). K = 1–15.
(TIF)

S3 Fig Results of best K selection from STRUCTURE for wild individuals only. A) Ln Pr(X|*K*). B) Delta *K*. C) Median of medians and means. D) Maximum of medians and means.
(TIFF)

S4 Fig CLUMPAK results of STRUCTURE run on wild individuals. K = 1–15.
(TIF)

S1 Table Genome assembly statistics for available full genomes of Nocutidae species. (XLSX)

S2 Table Number of individuals with private alleles within each population by locus. (XLSX)

S3 Table Results of the Hardy-Weinberg equilibrium test across individual loci within each population. (XLSX)

S4 Table Individual Sample ID, GPS coordinates (latitude, longitude), population number, site name, state/province and collection type. (XLSX)

S5 Table Filtered SNP dataset VCF file. (XLSX)

S6 Table Number of sites and mean depth of coverage per individual. (XLSX)
