# Supplementary figures and images for: Examining population structure of a bertha armyworm, Mamestra configurata (Lepidoptera: Noctuidae), outbreak in western North America: Implications for gene flow and dispersal
Source: PLoS One. 2019 Jun 27;14(6):e0218993. doi: 10.1371/journal.pone.0218993 (PMC6597092; doi:10.1371/journal.pone.0218993)

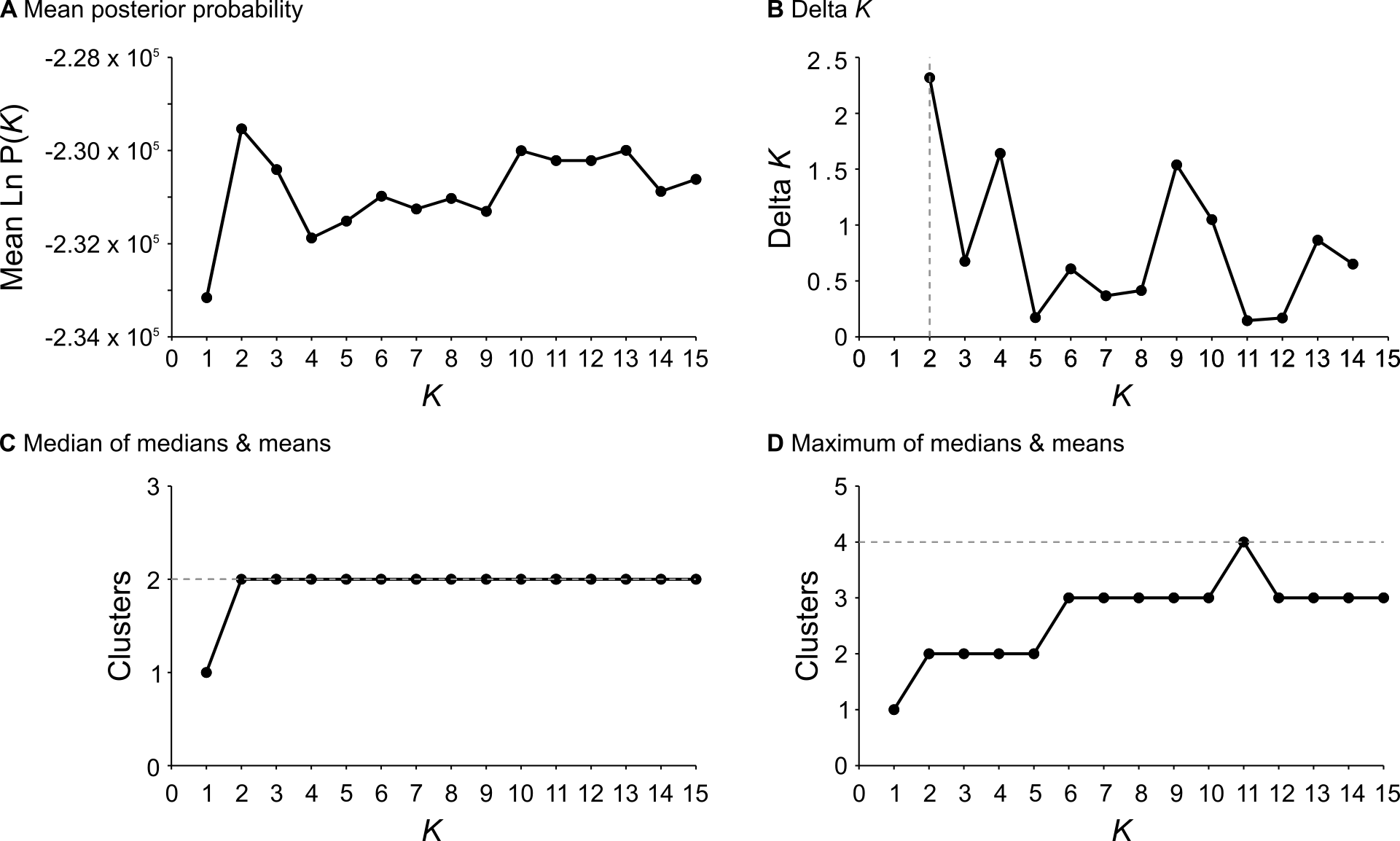

Supplement: S1 Fig — A) Ln Pr(X|K). B) Delta K. C) Median of medians and means. D) Maximum of medians and means. (TIFF) [file pone.0218993.s001.tiff]

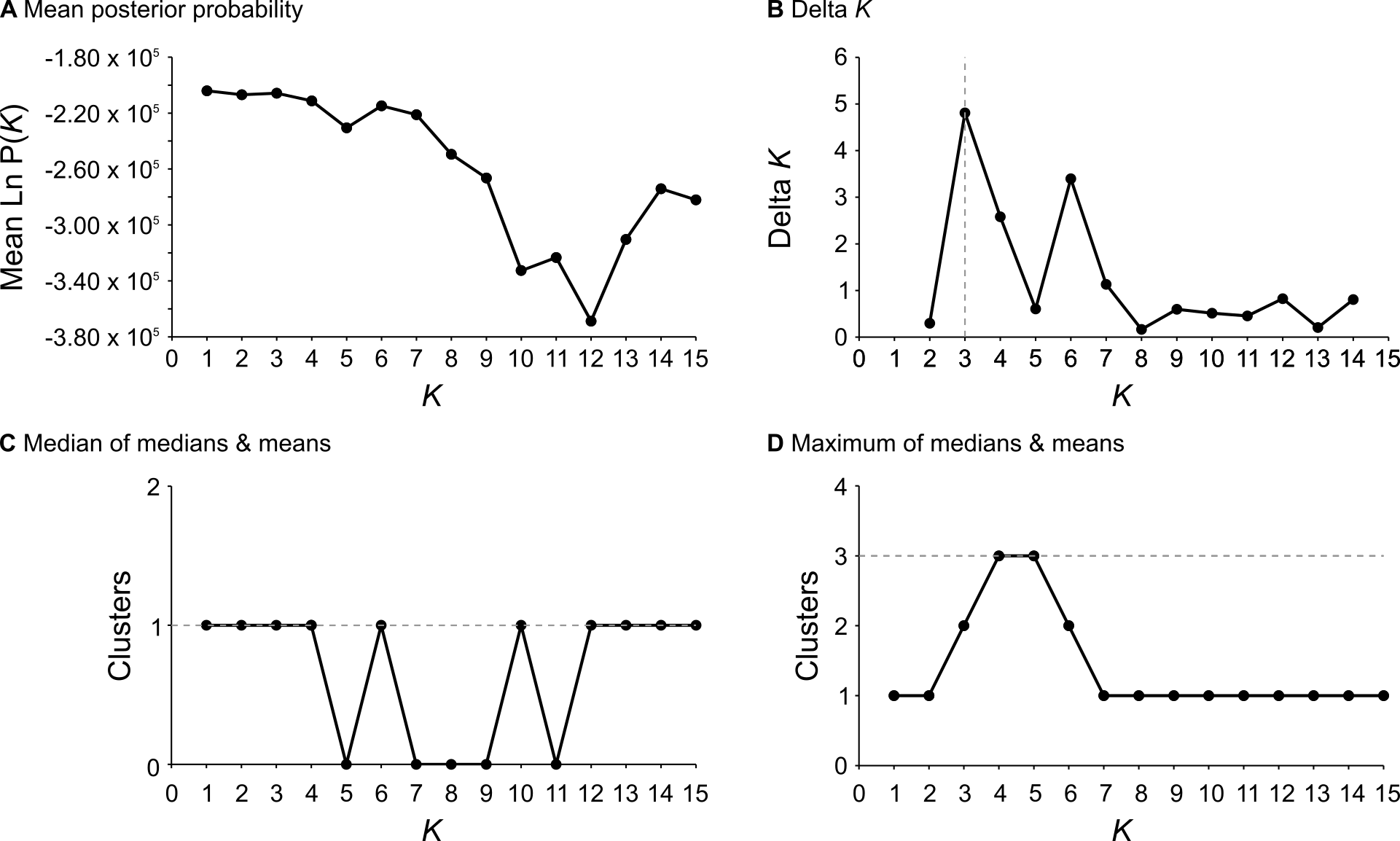

Supplement: S3 Fig — A) Ln Pr(X|K). B) Delta K. C) Median of medians and means. D) Maximum of medians and means. (TIFF) [file pone.0218993.s003.tiff]
